# Hungry neutrophils: platelet phagocytosis in EDTA-dependent pseudothrombocytopenia

**DOI:** 10.1016/j.htct.2026.108553

**Published:** 2026-10-15

**Authors:** Tiago Ascenção Barros, Matheus Vescovi Gonçalves

**Affiliations:** Grupo Fleury, São Paulo, SP, Brazil

A 29-year-old woman underwent routine complete blood count monitoring during levetiracetam therapy. Mild thrombocytopenia (136 × 10⁹/L) was detected, with normal hemoglobin and leukocyte counts. A peripheral blood smear revealed numerous neutrophils containing multiple intracytoplasmic platelets, with rare platelet satellitism (Figure 1). A repeat sample collected in sodium citrate showed a normal platelet count (243 × 10⁹/L), complete disappearance of the intracytoplasmic platelets, and rare residual satellitism (Figure 2), confirming EDTA-dependent pseudothrombocytopenia. This case illustrates platelet phagocytosis as an uncommon manifestation of EDTA-dependent pseudothrombocytopenia [1] and highlights the importance of the peripheral blood smear review to prevent diagnostic errors and unnecessary investigations.

**Figure 1:**
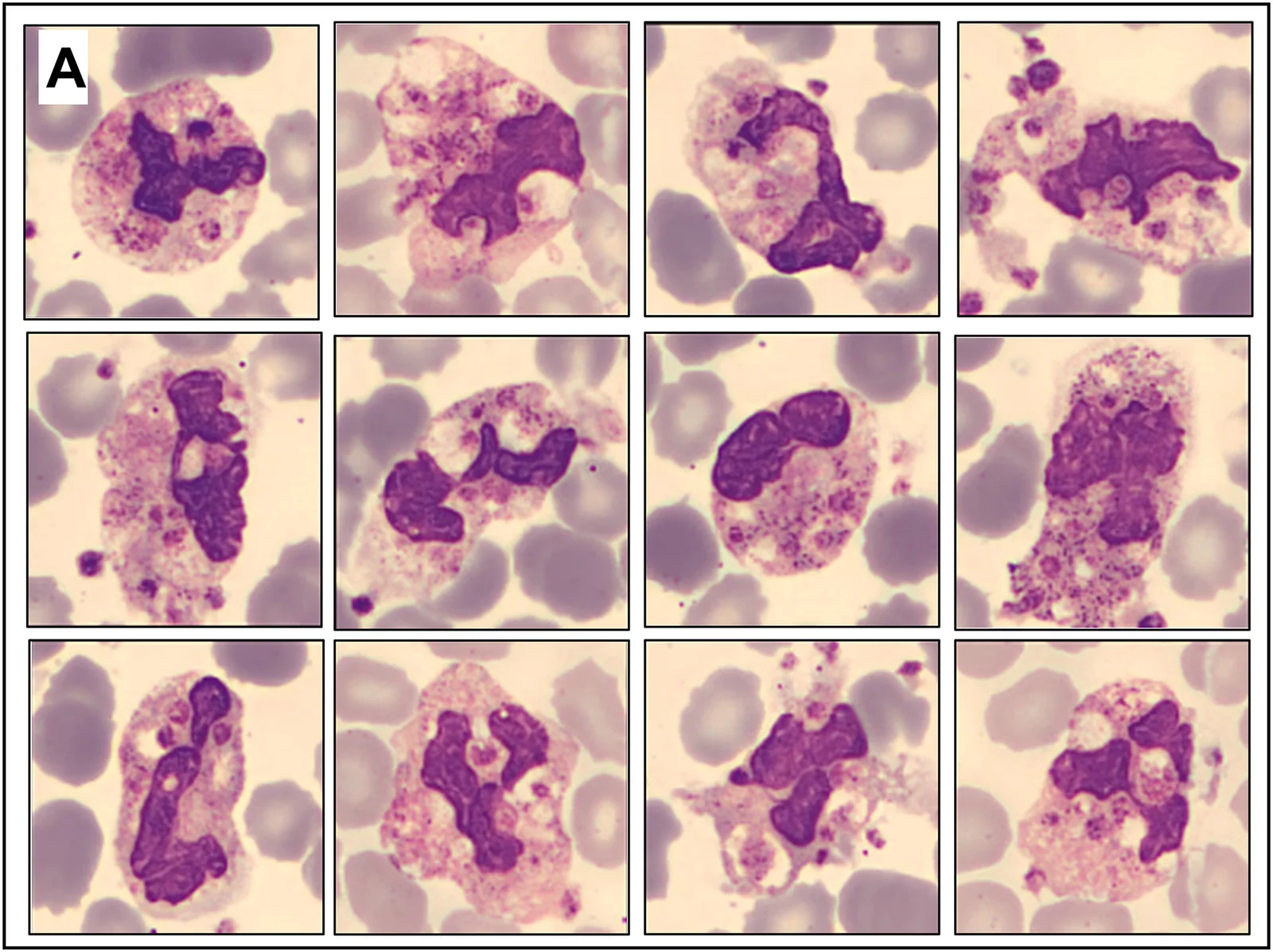
Peripheral blood smear from the EDTA sample showing multiple neutrophils containing intracytoplasmic platelets, with rare platelet satellitism. May-Grünwald-Giemsa stain; original magnification × 1000 (oil immersion).

**Figure 2:**
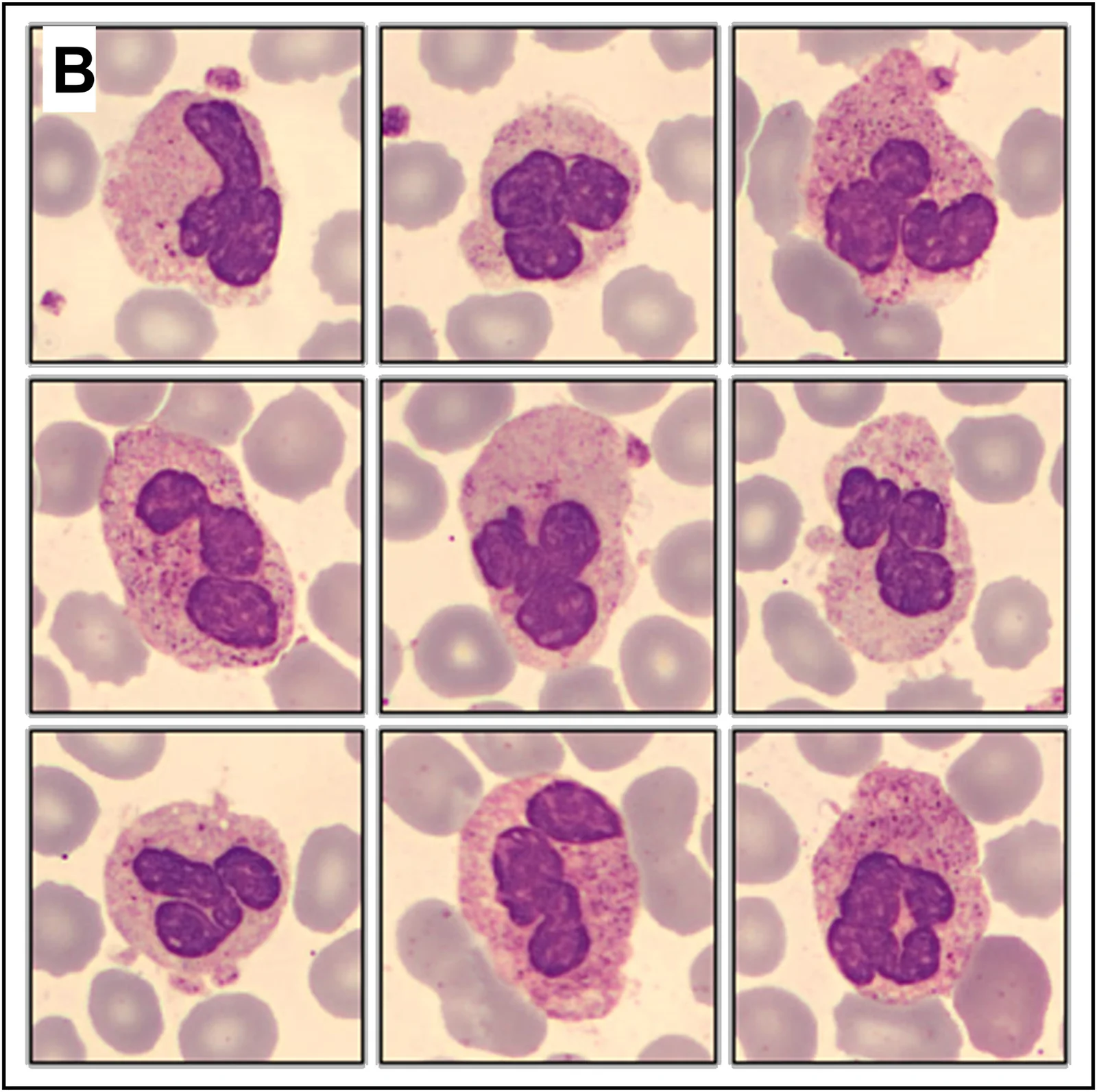
Peripheral blood smear from a sodium citrate sample obtained immediately afterward showing complete disappearance of intracytoplasmic platelets and only rare residual platelet satellitism, confirming EDTA-dependent pseudothrombocytopenia. May-Grünwald-Giemsa stain; original magnification × 1000 (oil immersion).

## Data availability

The data that support the findings of this study are available from the corresponding author upon reasonable request

## Conflicts of interest

The authors are employees of FLEURY S.A. The present work was conducted independently, and the employer had no role in the study design, data interpretation, manuscript preparation or the decision to submit it for publication.

## References

[1] Lippi G., Plebani M. (2012). EDTA-dependent pseudothrombocytopenia: further insights and recommendations for prevention of a clinically threatening artifact. Clin Chem Lab Med.

